# Validation of the Rasch-based Depression Screening in a large scale German general population sample

**DOI:** 10.1186/1477-7525-8-105

**Published:** 2010-09-21

**Authors:** Thomas Forkmann, Maren Boecker, Markus Wirtz, Heide Glaesmer, Elmar Brähler, Christine Norra, Siegfried Gauggel

**Affiliations:** 1 Institute of Medical Psychology and Medical Sociology, University Hospital of RWTH Aachen, Pauwelsstraße 30, 52074 Aachen, Germany; 2 Institute of Psychology, University of Education Freiburg, Kartäuserstr. 61b, 79117 Freiburg, Germany; 3 Department of Medical Psychology and Medical Sociology, University of Leipzig, Phillipp- Rosenthal-Straße 55, 04103 Leipzig, Germany; 4 Department of Psychiatry and Psychotherapy, LWL-University-Clinic, Ruhr-University Bochum, Alexandrinenstr. 1-3, 44791 Bochum, Germany

## Abstract

**Background:**

The study aimed at presenting normative data for both parallel forms of the "Rasch-based Depression Screening (DESC)", to examine its Rasch model conformity and convergent and divergent validity based on a representative sample of the German general population.

**Methods:**

The sample was selected with the assistance of a demographic consulting company applying a face to face interview (N = 2509; mean age = 49.4, SD = 18.2; 55.8% women). Adherence to Rasch model assumptions was determined with analysis of Rasch model fit (infit and outfit), unidimensionality, local independence (principal component factor analysis of the residuals, PCFAR) and differential item functioning (DIF) with regard to participants' age and gender. Norm values were calculated. Convergent and divergent validity was determined through intercorrelations with the depression and anxiety subscales of the Hospital Anxiety and Depression Scale (HADS-D and HADS-A).

**Results:**

Fit statistics were below critical values (< 1.3). There were no signs of DIF. The PCFAR revealed that the Rasch dimension "depression" explained 68.5% (DESC-I) and 69.3% (DESC-II) of the variance, respectively which suggests unidimensionality and local independence of the DESC. Correlations with HADS-D were *r_DESC-I _* = .61 and *r_DESC-II _* = .60, whereas correlations with HADS-A were *r_DESC-I _* = .62 and *r_DESC-II _* = .60.

**Conclusions:**

This study provided further support for the psychometric quality of the DESC. Both forms of the DESC adhered to Rasch model assumptions and showed intercorrelations with HADS subscales that are in line with the literature. The presented normative data offer important advancements for the interpretation of the questionnaire scores and enhance its usefulness for clinical and research applications.

## Background

Screening for depression is an important diagnostic task in many clinical settings. Several established screening instruments are available for this purpose like the Beck Depression Inventory [BDI; 1], the Patient Health Questionnaire 9 [PHQ-9; 2], or the Hospital Anxiety and Depression Scale [HADS; 3,4]. Most of the established instruments were originally developed on the basis of classical test theory (CTT) and many studies reported excellent reliability and validity for these instruments when relying upon CTT assumptions [e.g., 5,6].

However, in the last years it was demonstrated that diagnostic instruments could benefit substantially from modern statistical approaches like models of item response theory (IRT), e.g., the Rasch model. The Rasch model is one of the IRT models that holds some particularly beneficial attributes, e.g., interval scale level of model parameters, sample free test calibration, and item free person measurement [for an introduction to Rasch analysis, see 7,8]. Applying IRT techniques, a slightly more differentiated picture of the psychometric properties of the established screening instruments for depression emerged. For instance, by using IRT modeling it was shown that unidimensionality - an important aspect of test theory - cannot be taken for granted for some instruments [9,10]. Furthermore it was shown that instruments containing items related to somatic symptoms could lead to severe problems when assessing patients with comorbid somatic diseases. If patients suffering from a severe somatic illness reported somatic symptoms in a depression questionnaire those symptoms may be ascribed to the somatic ailment or a depressive episode [11-13]. This may lead to artificially increased depression scores. Moreover, using IRT methods it was shown that established questionnaires could be shortened without loss of information [14]. Generally, in many studies applying IRT techniques, sound psychometric characteristics of a depression screening instrument could only be found if at least some items were removed from the scale. The question, which items had to be removed largely depended on the sample investigated [e.g., 13,15-17]. However, sample dependent psychometric characteristics of screening instruments may aggravate the comparison of results across different samples or studies.

The Rasch-based **De**pression **Sc**reening (DESC) is one of the first instruments that were originally developed using Rasch analysis. Its development was motivated by two aspects. First, given the evidence for sample-dependency of psychometric characteristics of many screening instruments for depression when applying IRT modeling, the first aim was to use Rasch analysis to originally develop a new instrument with stable psychometric characteristics across a diversity of different clinical and non-clinical samples. Second, as prior studies have shown that using questionnaires of mood repeatedly at short intervals produces artificial alteration of sum scores [18,19] an instrument that provides two parallel forms was lacking. Parallel forms are beneficial for retest applications in longitudinal designs, e.g., monitoring symptom change across treatment.

The DESC has already been shown to fit the Rasch model in various patient samples, e.g., cardiologic, otorhinolaryngologic, neurologic patients or patients with mental illnesses [20,21]. So, research up to now suggests that the DESC is a psychometrically sound and concise screening instrument consisting of two parallel forms which measures depression severity across a broad range of depression severity with high test accuracy.

However, despite the development of the DESC is in an advanced stage, population based norms are lacking to date. Population based norms for the DESC would enhance easiness and reliability of diagnostic decisions based on the DESC sum score on a single case basis. It would provide important advancements for the interpretation of the questionnaire scores and enhance its usefulness for clinical and research applications.

The primary aim of the current study was therefore to collect normative data for both forms of the DESC based on a representative sample of the German general population. Prior to determination of norm values, Rasch model conformity of the DESC in this sample was examined. Furthermore, convergent and divergent validity of the DESC with regard to the anxiety and the depression subscale of the Hospital Anxiety and Depression Scale [HADS; 4,22] were determined. Possible applications of the presented normative data are discussed.

## Methods

### Sample

A representative sample of the German general population was selected with the assistance of a demographic consulting company (USUMA, Berlin, Germany). The area of Germany was divided into 258 sample areas representing the different regions of the country. In each sample area households were selected by using a random route procedure with start addresses. Beginning at the start address in an area, each 3^rd ^household was contacted in order to conduct a face to face interview. The sample was intended to be representative in terms of age, gender and education. Inclusion criteria were age at or above 14 years and German language skills (read and understand). Between May and July 2009, a total of 4,572 households (valid addresses only) were approached of which 2,524 agreed to participate (55.2%). If not at home a maximum of four attempts were made to contact the selected person. Twelve interviews were removed from the dataset because of incomplete questionnaires; demographic information of three persons was missing. Thus, the final study sample consisted of 2,509 persons. Mean age was 49.4 (SD = 18.2) with a range from 14 to 94 years. The majority (55.8%) were women. Sociodemographic characteristics of the sample are presented in table 1.

**Table 1:** Sample details

		Total N = 2509	Male 44.2% (N = 1109)	Female 55.8% (N = 1400)
**age**	M	49.4	48.8	50.0
	SD	18.2	18.1	18.4
**Urbanity**	Rural area	14.5% (363)	14.5% (203)	14.5% (160)
	Urban area	85.5 (2139)	85.5% (1195)	85.5 (944)
**Education**	No qualifications	1.8% (44)	2.5% (35)	0.8% (9)
	< 10 years	42.3% (1059)	42.5% (594)	42.1% (465)
	10 years of education	35.9% (898)	38.3% (536)	32.8% (362)
	> 10 years	16.8% (420)	14.3% (200)	19.9% (220)
**Net household income**	< 1250€/month	24.1% (603)	27.0% (377)	20.5% (226)
	1251 to 2500€/month	50.4% (1262)	48.9% (684)	52.4% (578)
	≥ 2500€/month	22.7% (567)	21.2% (296)	24.5% (271)

All participants were visited by an interview assistant and informed about the investigation. The interview was based on a structured questionnaire that was filled in by the respondents. An interview assistant waited until the participant completed all questions and offered help if participants did not understand the meaning of the questions or the use of the response scale. The study procedures were in accordance with the declaration of Helsinki and approved by the local ethics committee.

### Material

*DESC*. The Rasch-based Depression Screening [DESC; 20] was developed on the basis of a calibrated Rasch-homogeneous item bank [see 23 for details on the construction process]. For the development of the DESC, items of the item bank were selected if they showed an excellent fit to the Rasch model. Furthermore, selected items should capture a broad range of depression severity similar to the range covered by the whole item bank. Structural equation modelling was applied to evaluate equivalence of the two scales [20]. Using Receiver Operating Characteristics (ROC) curves analysis the optimal cut-off score of both DESC forms was determined to be ≥12 with regard to interview-based diagnosis of a depressive disorder according to ICD-10 [24]. This cut-off score proved to be sensitive and specific. The DESC was developed to assess depression in both patients with mental and somatic illnesses. In the initial development it was found that no items on somatic symptoms could be included to the instrument because these items did not fit the model and violated the unidimensionality assumption of the scale [20].

The DESC consists of two parallel versions with 10 items each. Items refer to the last two weeks, and participants are asked to mark how often they experienced each symptom on a 5-point Likert scale from 0 (never) to 4 (always). An example of a DESC item is "how often during the last two weeks did you feel sad?" (See table 2 for abbreviations of all DESC items). Total scores range from 0 to 40 with higher scores indicating greater depression. Participants completed both forms of the DESC. The DESC is available from the principal author.

**Table 2:** Item characteristics of the Rasch-based Depression Screening I (DESC-I) and the Rasch-based Depression Screening II (DESC-II)

Item	δ_i _*	S.E.	infit	Outfit
*Threshold*			*< 1.30*	* < 1.30*
DESC-I				
sad	-.85	.04	.93	1.01
lonely	-.62	.05	.78	.75
despaired	-.44	.04	.71	.65
hopeless	-.31	.05	.67	.65
empty	-.08	.05	.74	.84
loss of joy	.24	.05	.60	.57
feel superfluous	.31	.05	.67	.61
life is a burden	.53	.06	.75	.76
life is a failure	.68	.06	.66	.49
suicide	1.28	.09	.85	.67
DESC-II				
disheartened	-.80	.04	.86	.88
little pleasure	-.66	.04	.83	.85
withdrawal	-.33	.04	.92	.93
discouraged	-.25	.04	.68	.66
uninspired	-.03	.04	1.02	1.13
pessimistic	.20	.05	.65	.68
feel needless	.30	.05	.97	1.09
be no good	.60	.06	.67	.62
loss of interest	.89	.06	.86	.87
suicide	1.05	.08	.83	.52

Note: Measures *δ_i _*were anchored on the original calibration sample reported in Forkmann et al. [20]; S.E.: Standard Error

*HADS*. The Hospital Anxiety and Depression Scale [HADS; 3,4,25] refers to the last week and consists of 14 items which are Likert scaled from 0 to 3 with changing polarity. Seven items each constitute the anxiety and the depression subscales. A cut off score of ≥ 8 is recommended to identify persons suffering from a depressive disorder according to ICD-10 [26]. The HADS was used to calculate measures of convergent and divergent validity of the DESC. The HADS was chosen to validate the DESC because it was originally developed for depression screening in patients with somatic diseases, which is also one the main fields of application for the DESC. Furthermore, it provides screening information on depression and anxiety symptoms, so that both convergent and divergent validity could be examined simultaneously.

*Further material*. All participants completed a demographic data sheet.

### Data analysis

Data analysis was conducted in two steps. In the first step, it was examined whether the Rasch model holds in the representative German general population sample. In the second step, based on these data norm values and measures of convergent and divergent validity were determined.

#### Step 1: Rasch analysis

The Rasch model conceptualizes the probability that a person will endorse an item as a logistic function of the difference between the person's level of, in this case, depression (*θ*, also referred to as the latent trait score or person measure) and the level of depression expressed by the item (*δ_i_*) [27]. Because the Rasch model was originally developed for intelligence and attainment tests, *δ_i _* is also often referred to as "item difficulty" [27]. For self-report instruments, this term can be "translated" as probability expressed in logits to endorse a high category of an item. For "difficult" items this probability would be lower than for "easy" items, relative to the individual person measure. In this step, all analyses were performed applying an extension of the Rasch Model, the Partial Credit Model [PCM; 28]. The PCM allows response categories to vary across items. This model was chosen because it was shown to be more appropriate to use the PCM than the competing Rating Scale Model in the original development of the DESC [20]. To ensure comparability of the results presented here with the original development sample of the DESC, item difficulty estimates *δ_i _* and thresholds were anchored on the original calibration sample reported in Forkmann et al. [20].

#### Separation and reliability

The item and person separation indices estimate the spread or separation of persons and items on the measured variable relative to measurement error. Items must be sufficiently well separated in terms of item difficulty in order to identify the direction and meaning of the latent scale [29]. A clinically useful set of items should define at least three strata of patients and items (e.g., high, moderate, and low levels of symptom burden), which are reflected in a separation index of 2.0 and an associated separation reliability of. 80 [8,29].

#### Rasch model fit

Infit and outfit are mean square residual statistics of model fit discrepancy with an expectation of 1.0 and a range from 0 to infinity. Infit and outfit statistics reflect slightly different approaches to assessing the fit of an item: The infit statistic gives relatively more weight to the answers of those persons closer to the item measure, whereas the outfit statistic is not weighted and therefore more sensitive to the influence of "outlying", i.e. more extreme responses. Values ≤ 1.3 indicate good fit [7].

#### Unidimensionality and local independence

Unidimensionality and local independence are two important interrelated assumptions of Item Response Theory. Unidimensionality means that only one single latent dimension (e.g., depression) accounts for the common variance in the data. Evidence of essential unidimensionality provides support for the assumption of local independence because if all items measure the same underlying construct, this construct accounts for any relationships among items, and other relationships among items are unlikely [30]. Thus, local independence means that when controlling for the major latent dimension no substantial intercorrelations between the items shall remain. A principal component factor analysis of the residuals (PCFAR) was performed [31,32]. Since uniform criteria have yet to be established for when a potential additional dimension would have to be considered, results were interpreted according to the recommendations of Linacre [33]: > 60% of variance explained by the Rasch dimension and ≤ 5% explained by the greatest potential additional dimension was considered as good. Additionally, an eigenvalue ≤ 3 indicates that the potential second dimension has only marginal explanatory power. This result allows for ignoring further components [33].

#### Evaluation of Differential Item Functioning (DIF)

Differential item functioning (DIF) investigates the items in an instrument for signs of interactions with sample characteristics. DIF analyses were performed for gender and age for three reasons: Firstly, many studies showed that prevalence of depression depends on age and gender [34,35]. Thus, DIF due to these variables might be suspected. Secondly, prior studies analysing self-report instruments for depression found DIF related to age [36-38] and DIF related to gender [39]. Furthermore, we considered it most important to investigate whether the DESC can be used for both genders and all age groups without different norms because this would imply a notable practical advantage for clinical practice. Therefore, Item difficulty measures *δ_i _* were computed for each class of subjects (e.g., men vs. women) to be contrasted. A two-sided *t*-test was then performed pair wise comparing item difficulty measures for subject classes (*α *≤ 0.01). In accordance to the studies reporting the initial development of the DESC [20,23] and following Linacre's recommendations to interpret these *t*-tests conservatively, additionally to the significant *t*-test, a DIF contrast (i.e., DIF measure for subject class 1 minus subject class 2) of | > .5| was considered substantial [33].

#### Step 2: Determination of DESC norm values and measures of convergent and divergent validity

After determination of adherence to Rasch model assumptions norm values were calculated separately for DESC-I and DESC-II according to the following routine. First, based on the individual raw sum scores each person's latent trait score *θ *was calculated. Then, trait scores *θ *were transformed linearly into percentiles, z-values (mean = 0, SD = 1) and T-values (mean = 50, SD = 10). Afterwards, correlations of both DESC forms with the depression and the anxiety scale of the HADS were calculated as measures for convergent and divergent validity. Possible applications of these normative data for the assessment of change in clinical diagnostics are exemplified in the discussion section.

All analyses were conducted using WINSTEPS 3.60.1 and SPSS 17.

## Results

### Step 1: Rasch analysis

#### Separation and reliability

Item separation for DESC-I (11.15) and DESC-II (11.11) was very good as well as item reliability (DESC-I = .99; DESC-II = .99). Person separation (DESC-I = 1.51; DESC-II = 1.75) and person reliability (DESC-I = .69; DESC-II = .75) failed slightly the critical values. Cronbach's *α *was high with .92 for DESC-I and .93 for DESC-II, respectively.

#### Rasch model fit

All items of both DESC-I and DESC-II adhered to the infit and outfit criteria of < 1.3 indicating very good Rasch model fit. See table 2 for details.

#### Unidimensionality and local independence

To evaluate unidimensionality and local independence the residual correlation matrix was examined. A principal component factor analysis of the residuals (PCFAR) revealed that the Rasch dimension "depression" explained 68.5% of the variance (eigenvalue 21.8) in DESC-I and 69.3% of the variance (eigenvalue 22.6) in DESC-II. The biggest potential secondary dimension explained 5.0% of the variance (eigenvalue 1.6) both in DESC-I and DESC-II. This result is in line with the assumptions of both unidimensionality and local independence of the data, since the recommendations of Linacre [33] are fulfilled.

#### Evaluation of Differential Item Functioning (DIF)

There were no signs of DIF due to age or gender for both DESC-I and DESC-II. Thus, sum scores of both forms of DESC may be interpreted independently from the respondents' age or gender.

### Step 2: Determination of DESC norm values and measures of convergent and divergent validity

Since Rasch model conformity of both forms of the DESC could be confirmed in the present sample, norm values were determined applying the routine outlined above. Norms were not calculated separately for gender or different age groups, since Rasch analysis revealed that DESC sum-scores can be interpreted independently of age or gender. Norm values (percentiles, Z-, and T-scores) are presented in tables 3 and 4 together with raw scores and the Rasch measures θ.

**Table 3:** Norm values for DESC-I

raw score	frequency	percentage	percentiles	*θ*	Z	T
0	841	33.5	33.5	-5.80	-1.09	39
1	390	15.5	49.1	-4.52	-0.38	46
2	271	10.8	59.9	-3.71	0.07	51
3	199	7.9	67.8	-3.19	0.36	54
4	153	6.1	73.9	-2.78	0.59	56
5	126	5.0	78.9	-2.42	0.79	58
6	105	4.2	83.1	-2.10	0.97	60
7	66	2.6	85.7	-1.81	1.13	61
8	69	2.8	88.5	-1.53	1.29	63
9	61	2.4	90.9	-1.26	1.44	64
10	60	2.4	93.3	-1.00	1.58	66
11	59	2.4	95.7	-0.75	1.72	67
12	42	1.7	97.3	-0.51	1.86	69
13	14	.6	97.9	-0.28	1.99	70
14	11	.4	98.3	-0.06	2.11	71
15	11	.4	98.8	0.16	2.23	72
16	8	.3	99.1	0.37	2.35	73
17	6	.2	99.3	0.57	2.46	75
18	4	.2	99.5	0.78	2.58	76
19	3	.1	99.6	0.98	2.69	77
20	3	.1	99.7	1.18	2.80	78
21	2	.1	99.8	1.38	2.91	79
22	1	.0	99.8	1.59	3.03	80
23	1	.0	99.9	1.82	3.16	82
26	1	.0	99.9	2.61	3.60	86
27	1	.0	100.0	2.97	3.80	88
>/= 28	1	.0	100.0	3.45	4.07	91

Note: *θ*: estimated person's latent trait score for depression; Z: mean = 0, SD = 1; T: mean = 50, SD = 10

**Table 4:** Norm values for DESC-II

raw score	frequency	percentage	percentiles	*θ*	Z	T
0	963	38.4	38.4	-6.03	-1.02	40
1	273	10.9	49.3	-4.74	-0.36	46
2	244	9.7	59.0	-3.93	0.05	50
3	159	6.3	65.3	-3.41	0.31	53
4	129	5.1	70.5	-3.00	0.52	55
5	120	4.8	75.2	-2.66	0.70	57
6	106	4.2	79.5	-2.35	0.85	59
7	81	3.2	82.7	-2.08	0.99	60
8	58	2.3	85.0	-1.82	1.12	61
9	54	2.2	87.2	-1.58	1.25	62
10	55	2.2	89.4	-1.35	1.36	64
11	47	1.9	91.2	-1.13	1.48	65
12	37	1.5	92.7	-0.92	1.58	66
13	44	1.8	94.5	-0.71	1.69	67
14	37	1.5	95.9	-0.51	1.79	68
15	30	1.2	97.1	-0.31	1.89	69
16	17	.7	97.8	-0.11	1.99	70
17	17	.7	98.5	0.08	2.09	71
18	5	.2	98.7	0.27	2.19	72
19	6	.2	98.9	0.46	2.29	73
20	6	.2	99.2	0.65	2.38	74
21	3	.1	99.3	0.84	2.48	75
22	7	.3	99.6	1.03	2.58	76
23	2	.1	99.6	1.22	2.67	77
24	3	.1	99.8	1.41	2.77	78
25	1	.0	99.8	1.61	2.87	79
26	1	.0	99.8	1.82	2.98	80
27	2	.1	99.9	2.05	3.10	81
30	1	.0	100.0	2.86	3.51	85
>/= 31	1	.0	100.0	3.22	3.69	87

Note: *θ*: estimated person's latent trait score for depression; Z: mean = 0, SD = 1; T: mean = 50, SD = 10

The population mean of DESC-I was M = 3.9 (SD = 5.4) and of DESC-II was M = 4.0 (SD = 5.6). When applying the proposed cut-off score of 12 [20], DESC-I would classify 10.0% of the representative sample as being depressed, while DESC-II classifies 10.8% to be depressed. The concordance of both classifications according to the coefficient *κ *for nominal data is *κ *= .73. The depression subscale of the HADS would classify 24.5% of the sample as depressed.

The parallel test reliability of DESC-I and -II was *r *= .93 (p < .01). The correlation with the depression subscale of the HADS was moderate for DESC-I (*r *= .61; p < .01) as well as for DESC-II (*r *= .60). The correlation with the anxiety subscale of the HADS was *r *= .62 for DESC-I and *r *= .60 for DESC-II.

## Discussion

This study aimed at validating the DESC in a representative sample of the German general population and at providing normative data and measures of convergent and divergent validity of both forms of the instrument.

Overall, both forms of the DESC adhered to Rasch model assumptions. We found very good Rasch model fit according to infit and outfit statistics, strong evidence for unidimensionality and local independence, and no signs of differential item functioning. Keeping in mind that the DESC's validity in clinical samples has already been shown [20,40], these results additionally suggest, that the DESC appears to be a psychometrically sound instrument for screening for depression in the general population. Furthermore, the high parallel test reliability could be replicated indicating that the DESC can be applied as true parallel versions of the same inventory in retest applications.

The fraction of the sample that was classified as depressed when applying the proposed cut-off score of the two DESC parallel forms roughly corresponds to the German prevalence rates reported in the literature [see e.g., 41]. While sound criteria for external validity are lacking in the current study, this concordance may be cautiously interpreted as suggesting validity. Furthermore, prior studies in patient samples indicated good external validity of the DESC [see e.g., 20].

The reported values for convergent and divergent validity were moderate. Anxiety and depression are known to be substantially correlated so that moderate positive correlations of self-report instruments for depression with measures of anxiety are a common phenomenon. Thus, the moderate positive correlation with the anxiety subscale of the HADS is in concordance with prior literature [42]. Furthermore, the correlation between the depression and anxiety subscales of the HADS itself was comparably high (*r *= .68) so that the moderate positive correlation of the DESC with anxiety does not flaw its validity.

We expected high convergent validity with the depression subscale of the HADS. However, the revealed correlation was only moderate, too. In order to appraise the significance of this result for the standing of the DESC compared to the established self-report instruments for depression, like the HADS [4], the Beck Depression Inventory [BDI; 1], or the Center for Epidemiologic Studies Depression Scale [CES-D; 43], it has to be taken into account that moderate convergent validity with other self-report instruments for depression has been reported for most other depression questionnaires, too. For example, both Bonilla and colleagues [44] and Kojima and colleagues [45] reported a correlation between BDI and CES-D of *r *= .69. Cameron and colleagues [46] found a correlation between the HADS and the Patient Health Questionnaire [PHQ-9; 2] of *r *= .68. Thus, the correlation between DESC and the depression subscale of the HADS is in concordance with recent findings from the literature. Furthermore, HADS and DESC might emphasize different aspects of depression. For example, in contrast to the HADS both forms of the DESC contain an item about suicidal ideation and behaviour which could at least partly account for the surprising results. Moreover, DESC (2 weeks) and HADS (1 week) refer to different timeframes and the HADS contains items with changing polarity whereas the DESC does not. Theses factors might add to the relatively low correlation of the scales. Above, in our sample the HADS classified more then twice as many persons as depressed as the DESC. Since the DESC classifications roughly correspond to the prevalence of depression reported in the literature this result might be interpreted as indicating that the HADS tends to produce "false positives" in the general population - a fact that has already been discussed for depression screening with the HADS in other samples [e.g., 26]. Nevertheless, future research should further investigate the construct validity of the DESC to substantiate the present findings.

### Possible applications of the presented normative data

The DESC was shown to be a reliable and valid instrument in prior studies [20,40]. Its sum-score can be interpreted as valid quantitative estimate of a person's depressive symptom burden, and it provides a sensitive and specific cut-off score which aids in deciding whether a depressive disorder is likely to be present. However, the normative data presented in the current study further facilitate the clinical utilization of the instrument. The provided T- and Z-scores allow for comparing DESC sumscores with the distribution of sumscores in the general population. Thus, clinicians may now come to a rapid binary decision about the clinical status of a patient by applying the cut-off score. But above, a more fine graded evaluation of the patient's state is possible by comparing his scores with the distribution in the general population. This may be beneficial for clinical application, particularly in repeated assessments. The issue how to determine significant change across treatment has been subject to intense and vivid discourse in psychotherapy research in the past [see 47 for a review]. Important recommendations how to deal with this problem have been made by e.g. Jacobson and Truax [48]. Amongst other important suggestions, they point out that a central aspect of the evaluation of clinical significant change is the returning of the patient's score to the range of the mean plus one standard deviation of the general population distribution. This refers to the "cutoff point *b*" as presented by the authors [see 47 for details]. With the normative data presented here, clinicians now can follow this recommendation when using the DESC.

## Conclusions

Taken together, the present study provides further evidence for the psychometric quality of the DESC and opens new opportunities for sumscore interpretation through the presentation of normative data. The major strengths of the instrument can be expected in retest applications in both clinical and nonclinical samples. We conclude that the instrument can be useful in dealing with the central challenges of clinical assessment: (1) to measure a patient's symptom burden quantitatively, (2) to decide, whether this measurement indicates the presence of a depressive disorder, and (3) to judge whether symptom burden changes in the course of treatment.

## Competing interests

The authors declare that they have no competing interests.

## Authors' contributions

TF contributed to conception and design of the study, conducted the statistical analysis and wrote the manuscript. MB participated in the analysis and interpretation of the data. MW participated in the design of the study and the statistical analysis. HG and EB participated in the design of the study and coordinated the data acquisition. CN contributed to the analysis and interpretation of the data. SG have been involved in drafting and revising the manuscript, and coordinated the study and data acquisition. All authors read and approved the final manuscript.
